# THE CHALLENGING AND UNPREDICTABLE SPECTRUM OF COVID-19 IN CHILDREN AND ADOLESCENTS

**DOI:** 10.1590/1984-0462/2020/38/2020192

**Published:** 2020-09-07

**Authors:** Marco Aurelio Palazzi Safadi, Clovis Artur Almeida da Silva

**Affiliations:** a Faculdade de Ciências Médicas da Santa Casa de São Paulo, São Paulo, SP, Brazil.; b Universidade de São Paulo, São Paulo, SP, Brazil.

A novel coronavirus, named severe acute respiratory syndrome coronavirus 2 (SARS-CoV-2), emerged in China in the end of 2019 and after less than 6 months its related disease (COVID-19) has already affected more than 6 million individuals in almost all countries worldwide. COVID-19 was declared a pandemic by the World Health Organization on March 11, 2020, becoming one of the most challenging and concerning public health crisis faced by this generation.^1^ ^,^ ^2^ ^,^ ^3^ ^,^ ^4^

A striking feature of COVID-19 pandemic is that children and adolescents seem to be less frequently infected by SARS-CoV-2 comparing to adults. Preliminary evidence also shows that, unlike influenza or respiratory syncytial virus, children do not play a critical role in SARS-CoV-2 transmission in the community.^5^ Furthermore, although most infected children and adolescents are asymptomatic or present mild symptoms, recent unexpected data showing the emergence of a late-onset severe inflammatory syndrome temporally associated with SARS-CoV-2 highlights the importance of continued surveillance around the world.^6^

Data from laboratory-confirmed COVID-19 cases in Asia, Europe and North America, by age groups, showed that the prevalence of children and adolescents in these case series ranged from 1.0 to 1.7%. The clinical spectrum of pediatric COVID-19 is wide, ranging from asymptomatic to critically ill cases. Fever and cough were consistently the most common reported symptoms in these case series, although less frequently than in adults, followed by pharyngeal erythema, shortness of breath, rhinorrhea, nausea, abdominal pain, vomiting and diarrhea. Additional symptoms reported included myalgia, tiredness, headache, anosmia and ageusia. More recently, variable cutaneous manifestations have been described in pediatric populations with COVID-19, including erythematous rashes, urticaria, vesicular and chilblain-like lesions.^7^

Leucopenia, lymphopenia and increased inflammatory markers (erythrocyte sedimentation rate, C-reactive protein or procalcitonin) were the most frequently reported laboratorial findings in children and adolescents with COVID-19. Although data is limited comparing to adults, lymphopenia, high levels of C-reactive protein, procalcitonin, D-dimer and creatine kinase muscle and brain (MB) biomarkers were laboratorial findings associated with more severe disease.^1^ ^,^ ^2^ ^,^ ^3^ ^,^ ^4^ ^,^ ^5^ ^,^ ^6^ ^,^ ^7^

Clinical course of COVID-19 in children and adolescents uncommonly resulted in life-threatening illness with severe outcomes. In the largest reported case series from USA, with information on hospitalization status, approximately 20% of the children and adolescents were hospitalized and 2% of them were admitted in Pediatric Intensive Care Units (PICU). Importantly, infants aged <1 year represented the age group with the highest percentage of hospitalization among COVID-19 pediatric patients. Less than 1% of children and adolescents had severe COVID-19 with acute respiratory distress syndrome or multiorgan failure.^8^

A recent study reporting the outcomes of children and adolescents with COVID-19 admitted to USA and Canadian PICU showed severe disease is less frequent, and early outcomes in children hospitalized are far better comparing to adults. Interestingly, among 46 children and adolescents (median age 13 years) admitted to the PICU, 40 (83%) were found to have chronic underlying health conditions, 18 (38%) of them required invasive ventilatory support and only 2 (4.2%) died.^9^

In the end of Abril, cases of a severe rare syndrome, temporally associated with COVID-19, have been reported in children and adolescents, initially in Europe and then in North and Latin America. This syndrome, named multisystem inflammatory syndrome in children (MIS-C), occurs days to weeks after acute SARS-CoV-2 infection. The clinical characteristics of MIS-C share similar features with Kawasaki disease (KD), KD shock syndrome, macrophage activation syndrome (MAS) and toxic shock syndrome. Although many patients with MIS-C meet criteria for complete or incomplete KD, affected children were older, presented more intense inflammation and higher levels of markers of cardiac injury. A broad spectrum of presenting signs and symptoms and disease severity were observed among reported MIS-C cases, including persistent fever, gastrointestinal symptoms (abdominal pain, vomiting, diarrhea), rash, conjunctivitis, progressing in some cases to shock, myocarditis, acute heart failure and development of coronary artery aneurysms. Patients who have presented with this syndrome were, in general, previously healthy, and most of them have tested negative for SARS-CoV-2 RNA but positive for antibodies, suggesting an unbalanced immune response following SARS-CoV-2 infection. Laboratory findings include lymphocytopenia, increased inflammatory (C-reactive protein, erythrocyte sedimentation rate, D-dimer, ferritin) and cardiac biomarkers (troponin, brain natriuretic peptide [BNP]).^6^

Based on current evidence, older adults and people of all ages with underlying medical conditions, including severe obesity, chronic lung disease, cardiovascular disease, diabetes mellitus, chronic kidney disease, liver disease, active cancer, transplantation and immunocompromised have been associated with poor clinical outcomes and higher fatality rates from COVID-19.^1^ ^,^ ^3^

There are limited data on which underlying conditions in children and adolescents are associated with increased risk of infection or severe illness. Infants <1 year of age and children with chronic pulmonary diseases (including moderate to severe asthma), cardiovascular illnesses (including congenital heart disease), malignancy, immunosuppression and obesity appear to be at increased risk of severe disease.^8^ ^,^ ^9^ ^,^ ^10^

Data of immunocompromised patients with autoimmune diseases and COVID-19 are scarce. Although the true risk of life-threatening complications of this emerging infectious disease for these chronic illnesses is not yet known, there are particular concerns regarding SARS-CoV-2 infection for patients treated with immunosuppressive, biological agents and disease-modifying antirheumatic drugs.^11^

One of the most important Latin American reference centers for pediatric liver diseases and pediatric liver transplantation in Brazil described their experience with 169 non-transplant children and adolescents suspected and tested for SARS-CoV-2. Of note, 13/169 (8%) of them had laboratory-confirmed COVID-19. All of them had mild COVID-19, except one that died due to a serious genetic syndrome. Furthermore, during the study period, none of 190 pediatric liver transplant patients had COVID-19.^12^

Overall morbidity and mortality of COVID-19 in pediatric patients with cancer seem to be low. One of the largest pediatric cancer programs in the USA, in New York city, reported that 20/178 (11%) children and adolescents with cancer had positive test for SARS-CoV-2. Only one patient with COVID-19 required noncritical hospitalization.^13^ Malignancy in pediatric populations are generally aggressive, needing multiple chemotherapy or stem cell transplantation. Therefore, postponing these therapies are not recommended during COVID-19 pandemic.

Importantly, the long-term effects of this pandemic, with school closure and social isolation during quarantine/lockdown for children and adolescents, may influence sedentary behavior and consumption of calories-dense comfort foods, increasing the risk of weight gain and contributing for metabolic and cardiovascular diseases, particularly among those living in urban districts.^11^

There are other challenges related to this pandemic in children and adolescents. Non-pharmacological interventions have been an essential preventive measure, recommended by national and international public health authorities. Besides the risk of limited or even no education for children and adolescents during COVID-19 crisis, home confinements may induce longer screen time, physical inactivity, sleep abnormalities, increase alcohol intake risk and domestic violence, particularly in adolescents. Drug adherence should also be reinforced for patients with preexisting chronic disease and their families due to risk of disease flare or disease damage. Patients with suspected or confirmed COVID-19 must be strictly monitored for the possible risk of disease reactivation after the resolution of this viral infection.^11^

The overwhelmed public health systems by the COVID-19 pandemic represents a serious risk for pediatric general health, limiting access of children and adolescents to basic health care, compromising immunization coverages and postponing consultations for patients with underlying conditions. Moreover, mental health burden and socio-economic issues may contribute for short and long-term negative outcomes in children and adolescents and their families. Acute stress, anxiety, mild to severe depression, post-traumatic stress disorder, and emotional exhaustion may be first diagnosed during or after COVID-19 pandemic. Thus, online mental health care delivery, using teleconsultation or telephone support lines, may be required for pediatric populations.^14^

Identification of a safe and effective antiviral therapy, that could improve disease outcomes, has been object of extensive research worldwide. However, so far there are no convincing data showing that any of the several antivirals (protease inhibitor lopinavir/ritonavir, remdesivir or favipiravir) that are being tested proved to be safe and efficacious against SARS-CoV-2. Moreover, it must be acknowledged that the majority of these trials have been performed in adults, with very limited data, if any, for most of the different candidate antiviral therapies in children.^15^

It is also of paramount importance to have in mind that the overwhelming majority of children and adolescents with COVID-19, once infected, will develop a mild, self-limited form of disease. It means that a large number of patients would have to be treated in order to demonstrate the benefits of an antiviral, raising concerns of the potential adverse events associated with this intervention. This way, it is our opinion that, given the lack of evidence supporting safety and efficacy of the current available drugs for the treatment of COVID-19 in children and adolescents, only supportive care should be routinely recommended for the majority of cases. In selected cases, of severe disease presentations or potential risk for disease progression due to the presence of strong risk factors, the use of antiviral therapy might be considered on a case-by-case individualized decision, assuming that the benefits outweigh risks of potential adverse events of the drug used. It is recommended that, ideally, these off-label antiviral therapies for COVID-19 should occur as part of a clinical trial.

Post-exposure prophylaxis is another potential strategy for using antiviral therapies. In this context, a recent double-blind randomized trial tested the use of hydroxychloroquine within four days after the reported exposure. However, results did not show any effect of this drug on the prevention of illness compatible with COVID-19 or confirmed SARS-CoV-2 infection when used as post-exposure prophylaxis.^16^

The most exciting and fascinating chapter of the battle against COVID-19 is undoubtedly the development of a safe and effective vaccine. We currently have more than 130 candidate vaccines being developed, at least 10 of them already being tested in humans, using different vaccine platforms, including nucleic acid-based (mRNA and DNA), vector-based, and inactivated or recombinant protein vaccines. Studies performed with several vaccine strategies against the other zoonotic coronavirus, SARS-CoV and MERS-CoV, focused on the S protein target, paved the way to facilitate a more rapid development of the current SARS-CoV-2 vaccine.^17^

Although significant progress has been made in a very short period of time, we still have several unanswered questions and challenges to the development of a vaccine against SARS-CoV-2, including the theoretical risk of Antibody-Dependent-Enhancement, the lack of clear correlates of protection, the long-term persistence of the immune responses induced by vaccination, the number of vaccine doses required for different age groups, the probable need of adjuvants to trigger TH1 response, and high neutralizing antibodies to spare antigen dosing. It is also difficult to anticipate whether these vaccines will provide protection against infection (which would also have the possibility to decrease transmission in the community once high coverage is achieved) or only prevent disease severity and/or death.^17^

The role of a recent Bacille-Calmette-Guerin (BCG) immunization in the prevention of COVID-19 is also being investigated in clinical trials. Previous studies have shown that BCG immunization, besides its specific effect against severe forms of tuberculosis, induces a nonspecific protective immune response against other infections.^17^

In conclusion, almost six months into the COVID-19 pandemic, its epicenter has displaced from China, Europe and USA to Brazil, exposing our vulnerable population to devastating consequences. Despite the fact that children and adolescents appear to have lower prevalence, milder clinical manifestations and lower fatality rates, compared to other age groups, COVID-19 global crisis has a potentially profound, long-term negative impact on pediatric populations. The recent identification of rare and severe inflammatory syndrome cases of COVID-19 in older children and adolescents highlights its unpredictable pathogenesis spectrum and outcomes. Mental health burden, social impact and financial loss are important challenges for children and adolescents of this and future generations. Further multicenter and longitudinal pediatrics studies with large populations will be necessary to clarify these findings and to evaluate specific healthy and preexisting chronic diseases in children and adolescents.
